# Effect of Culture System on Developmental Competence, Cryosurvival and DNA-Fragmentation of In Vitro Bovine Blastocysts 

**Published:** 2011-03-21

**Authors:** Mahdi Hajian, Seyed Morteza Hosseini, Vajiheh Asgari, Somayyeh Ostadhoosseini, Mohsen Forouzanfar, Mohammad Hossein Nasr Esfahani

**Affiliations:** 1 Reproduction and Development Department, Reproductive Biomedicine Center, Royan Institute for Animal Biotechnology, ACECR, Isfahan, Iran; 2 Embryology Department, Royan Institute for Reproductive Biomedicine, ACECR, Tehran, Iran; 3 Animal Sciences Department, Islamic Azad University, Marvdasht Branch, Marvdasht, Iran

**Keywords:** Blastocyst, Vitrification, Cryoperservation, Embryo Development, Co-Culture

## Abstract

**Background:**

This study investigated the effect of two in vitro embryo culture systems (co-culture system versus cell-free sequential-media) on developmental competence, cryosurvival and DNA- fragmentation of in vitro developed bovine blastocysts.

**Materials and Methods:**

Bovine presumptive zygotes were cultured in Ménézo's B2 (B2) plus vero-cells or sequential synthetic oviductal fluid (SOF) for eight days. Subsequently, half of the expanded blastocysts developed in both groups were vitrified, warmed within 30 minutes and post- warming embryos along with their corresponding non-vitrified embryos were cultured for two additional days in the same medium used before vitrification. Embryo development, cryosurvival and apoptosis were compared between the groups.

**Results:**

For non-vitrified embryos, culture in SOF significantly promoted the potency of embryos to develop into blastocysts compared with the co-culture system. The difference in post vitrification survival rate of SOF blastocysts (83.3%) was insignificant compared with co-culture (84.3%). However, while total cell number of warmed blastocysts in the co-culture system was significantly higher in the co-culture versus the sequential system (215.4 vs. 170.4), the quality of survived embryos in terms of hatching ability and apoptosis was adversely affected by co-culture compared with SOF (65.0% vs. 74.3%, and 13.5% vs. 10.0%, respectively; p<0.05).

**Conclusion:**

Although co-culture system may increase the viability of embryos following cryopreservation, the potency and dynamics of blastocyst formation significantly increased with sequential media compared to the co-culture system which can compensate for the lower efficiency of sequential media for vitrification/warming purposes.

## Introduction

During the last decade, development of cell-free sequential media for the culture of mammalian preimplantation embryos has been an active, and albeit successful, area of research with comparable results with the co-culture system (1-5). However, a survey of literature indicates that the cryo-withstand of embryos developed under semi-defined or defined culture conditions are frequently lower than those reported for embryos co-cultured over helper cells (1, 4-8). Consequently, to obtain more appropriate vitrification results, some investigators have resorted to the use of somatic cell co-culture, cell-conditioned or complex media either during embryo development (before cryopreservation) or more commonly after cryopreservation (3,6, 8).

Although co-culture systems produce good developmental results as measured by both the frequency of blastocyst formation, particularly cryo-resistance (6-9), addition of helper cells to the culture medium not only has numerous drawbacks arising from cells but also carries a certain risk of infecting the embryos with pathogens and long term sequels (5, 8, 10). On the other hand, despite a great number of studies investigating the efficiency of different embryo culture systems in relation with the cryo-withstand (3, 7, 9, 11-20), limited studies have been performed that compare the efficiency of co-culture with sequential systems. Such studies would be of fundamental importance to reach a conclusion on the superiority of conventional cell-supported or recently developed cell-devoid embryo culture systems, particularly when there is a demand for cryopreservation.

During a recent study, we optimized a vitrification procedure with an embryo co-culture system that enabled vitrified bovine embryos to re-expand and hatch at rates similar to non-vitrified embryos (9). We designed this study, as the second step, to compare developmental competence, cryosurvival and DNA fragmentation of bovine zygotes cultured in the co-culture system with those developed under cell-free sequential conditions.

## Materials and Methods

This study was approved by the Ethical Committee of Royan Institute. Unless otherwise specified, chemicals and media were purchased from Sigma (St. Louis, MO, USA) and Gibco (Life Technologies, Rockville, MD, USA) companies, respectively.

### In vitro embryo production

In vitro maturation and fertilization of abattoir-derived bovine oocytes were carried out as described elsewhere (10). At 18-24 hours post insemination (pi), presumptive zygotes were dissected from surrounding cumulus cells and then cultured in either a co-culture system of vero cells in B2 medium (CCD, Paris, France) containing 10% fetal calf serum (FCS) or sequential synthetic oviductal fluid media (SOF1 and 2) as described by Thompson et al. (21). For each experiment, embryos were developed in 50 μl drops in 60 mm plates covered with 10 ml of paraffin oil. Embryos (10/50 μl droplets) were first cultured for 72 hours (phase 1) when the ratios of cleavage, 8-16 and morula were recorded and then refreshed into new culture droplets each 48 hours until day 8 (phase 2) when the ratios of days 6-7-8 blastulation were compared. On day 8 pi, good quality grades 1 and 2 blastocysts developed in both sequential SOF and B2 co-culture systems were selected and counted. Half of these embryos were randomly cultured until hatching for the following quality assessments: total cell number (TCN), viability assay and TUNEL measurement of DNA fragmentation. Meanwhile, the remaining half of the expanded blastocysts in both groups were removed from culture medium, vitrified, thawed within 30 minutes and further cultured for two additional days. The ratios of hatching/ degeneration of non-vitrified embryos along with the ratios of immediate/total degeneration, re-expansion and hatchability of vitrified embryos developed under different culture conditions were determined. Hatched embryos were used for quality assay as mentioned for non-vitrified embryos.

### Vitrification of embryos

The vitrification protocol used in this study was a modified technique of Martínez et al. (22) carried out as described previously (16). Briefly, embryos were pre-equilibrated in a solution of 7.5% ethylene glycol (EG) + 7.5% dimethyl sulfoxide (DMSO) in Dulbecco Modified Eagles' Medium (DMEM) supplemented with 20% FCS until the expanded blastocysts endured a period of shrinkage and returning (up to 8 minutes, at 38.5°C). Equilibrated embryos were then exposed to a vitrification solution consisting of 15% EG + 15% DMSO in holding medium (DMEM-F12 with 20% FCS) at room temperature for one min and then loaded into the tips of the cryotops (Cryologic; CVM™, Fibreplug & Sleeve, Australia) with a minimum amount of the vitrification solution and immediately plunged into liquid nitrogen (LN2). For thawing, embryos were removed from LN2 and quickly exposed to DMEM + 20% FCS supplemented with 1 M sucrose for 1 minutes on a warm plate (38.5°C). Then, embryos were transferred to DMEM + 20% FCS containing 0.25 M sucrose for 5 min and finally washed thoroughly in DMEM + 20%FCS. Viable embryos in each group were cultured in the corresponding culture system. The percentages of re-expansion, hatching and degeneration were determined at 12 hours and 48 hours post-thawing and hatched embryos were used for assessment of TCN, viability and DNA fragmentation.

### Assessments

The viability of hatched blastocysts developed in vitrified and non-vitrified groups was determined by the differential staining method of Hosseini et al. (23). In brief, hatched blastocysts were incubated for 30 minutes in pre-equilibrated culture medium containing propidium iodide (PI; 300 μg/ml) and bisbenzimide (Hoechst: H33342; 5 μg/ml). Embryos were then washed in phosphate buffer saline (PBS) free of calcium and magnesium and then fixed in 2.5% glutharaldehyde. Fixed embryos were then washed in PBS before being mounted on microscopic slides. The stained embryos were examined under an epifluorescent microscope [excitation wavelength of 330-385 nm and barrier filter (400 nm)] to detect necrosed versus viable blastomers as red and blue in appearance, respectively. Moreover, determination of DNA-fragmentation within the embryonic mass was performed by Hosseini et al. (9).

### Statistical analysis

All experiments in this study were repeated at least three times. To compare rates of blastocyst development and hatching between treatments, chi square analysis was applied. The analysis of variance (ANOVA) was used for vitrification groups and the t test was used for comparisons of means. When the ANOVA test showed statistical differences, the Student- Neumann-Keuls test was used to discriminate between groups. All statistical evaluations were carried out using the Statistical Package for Social Sciences (SPSS), version 11. All data were presented as means ± SEM and differences were significant at p<0.05.

## Results

### Effect of culture system on the yield and quality of in vitro embryo production

From 420 bovine ovaries obtained from an abattoir, 2540 COCs were cultured for in vitro maturation (IVM), 2230 were subjected to in vitro fertilization (IVF) and 1840 presumptive zygotes were subsequently cultured in sequential SOF (n=900) and vero B2 co-culture systems (n=1250) for 8 days when the ratios of cleavage, morula, and days 6-8 blastocyst formation were determined. Table 1 compares detailed results of developmental competence in addition to the quality of the resultant blastocysts developed under both culture systems. As shown in Table 1, there was no significant difference in cleavage rate of embryos cultured in the co-culture (92.0%) versus SOF (86.5%) groups (p<0.05). However, zygotic development in the co-culture conditions resulted in significantly greater development to the 8-16 (87.0%) and morula (75.0%) stages compared to SOF culture media (47.3% and 54.4%, respectively; p<0.05). On the other hand, while 37.1% of morula stage embryos in SOF medium developed to blastocysts at day 6 of in vitro production (IVP), none of the embryos (0.0%) in the co-culture system developed into blastocysts at the same day. Similarly, the blastocyst yield rate at days 7 and 8 were significantly higher when embryo development occurred in SOF versus the coculture system (49.3% and 51.2% vs. 28.3% and 40.1%, respectively; p<0.05; Table 1).

Hatching ability of the blastocysts in both groups were non-significantly different (p<0.05). Comparing the quality of hatched blastocysts developed in the two culture systems, although the culture upon monolayer significantly increased total cell number (TCN) of the blastocysts that developed compared to SOF (248.0 vs. 221.0), the inner cell mass / trophectoderm (ICM/TE) ratio of the SOF-cultured embryos (41.5%) was significantly higher than the related rate of co-cultured embryos (33.1%). However, there was no significant difference in the viability and DNA fragmentation index (DFI) of the blastocysts developed in both groups.

### Effect of culture system on post-warming survival of the vitrified blastocysts

After vitrification/warming of co-culture versus SOF developed blastocysts, there was no significant difference in the proportion of immediate degenerated blastocysts (15.7% vs. 16.7%) nor the proportion of embryos re-expanded when assessed 12 hours post warming (84.3% vs. 83.3%; p<0.05). However, the hatching rate of vitrified/ warmed blastocysts developed in SOF medium (65.0%) was significantly lower than the rate of the co-culture condition (74.3%). Consequently, the post-warming total degeneration rate of the coculture group (25.7%) was significantly lower than the SOF (35.0%) group.

After vitrification-warming, blastocysts developed in SOF medium had signifcantly lower quality in terms of TCN and ICM/TE ratio than those developed in the co-culture system. Although analysis of viability indicated no significant difference between the hatched blastocysts of the two groups, DFI value of the co-culture group (10.0%) was significantly lower than the SOF group (13.5%).

**Table 1:** Effect of culture system on developmental yield and quality of in vitro produced bovine embryos


		Numbers (%)^1^ of embryos developed to:										
		Blastocyst							Blastocyst quality			

**IVP culture system**	**Presumptive zygotes (n)**	**Cleavage**	**8-16**	**Morula**	**D6**	**D7**	**D8**	**Hatched**	**TCN (n)**	**ICM/TE (%)**	**Viability^2^**	**DFI^3^(%)**
**Co-culture**	1250	1150(92.0 ± 3.1)	1000 (87.0 ± 2.3)*	862(75.0 ± 3.1)*	0(0.0 ± 0.0)*	325(28.3 ± 2.2)*	461 (40.1±1.1)*	384 (83.3 ± 3.6)	248.0 ± 4.5	33.1 ± 1.1*	96.3 ± 0.3	6.1 ± 0.0
**SOF**	885	765(86.5 ± 4.3)	362 (47.3 ± 2.0)*	416 (54.4 ± 4.1)*	284 (37.1 ± 3.9)*	377 (49.3 ± 3.2)*	391 (51.2±3.2)*	316(80.8 ± 3.1)	221.0 ± 4.5	41.5 ± 2.5*	94.7 ± 0.5	5.5 ± 0.7


1. Cleavage rates expressed based on the number of presumptive zygotes; the percentages of embryos that further progressed to blastocysts were expressed based on the number of embryos cleaved and the percentages of hatching were expressed based on the total number of blastocysts. 2. Percentage of live cells/TCN. 3. DFI: DNA fragmentation index, expressed as the percentage of TUNEL-positive/TCN cell nuclei. *indicates significant difference between the two groups (p<0.05). All percentages expressed as mean ± SEM.

**Table 2:** Effect of culture system on cryosurvival and post-warming quality of vitrified bovine blastocysts produced in vitro


						Blastocysts quality			

IVP Culturesystem	Vitrified embryos(n)	Immediate degeneration (%)	Post-warming re-expansion after12 hours (%)	Total hatched	Totaldegeneration	TCN(n)	ICM/TE(%)	Viability^1^ (%)	DFI^2^(%)	
**Co-culture**	96	15(15.7 ± 1.5)	81(84.3 ± 1.6)	60 (74.3 ± 2.0)*	40 (25.7 ± 3.0)*	215.4 ± 4.5*	36.0 ± 2.2*	95.2 ± 1.0	10.0 ± 1.0*	
**SOF**	122	21 (16.7 ± 1.0)	101 (83.3 ± 1.0)	65 (65.0 ± 2.4)*	36 (35.0 ± 2.5)*	170.4 ± 4.2*	44.6 ± 2.3*	93.4 ± 0.0	13.5 ± 1.5 *	


1. Percent of live cells/TCN. 2. DFI: DNA fragmentation index, expressed as the percentage of TUNEL-positive/TCN cell nuclei. *Indicates significant difference between the two groups (p<0.05). All percentages expressed as mean ± SEM.

**Table 3:** Comparison between different cryosurvival parameters of vitrified vs. non-vitrified embryos cultured under different culture conditions


	Comparisons														
IVP culture system	Hatching (%)			TCN (n)			ICM/TE (%)			Viability (%)			DFI (%)ICM/TE (%)		

	**Values (V vs. NV)**		**LD#**	**Values (V vs. NV)**		**LD**	**Values (V vs. NV)**		**LD**	**Values (V vs. NV)**		**LD**	**Values (V vs. NV)**		**LD**
**Co-culture**	74.3 ± 2.0vs.	83.3 ± 3.6*	9.0 ± 1.6^a^	215.4 ± 4.5vs.	248.0 ± 4.5*	77.6 ± 0.3^a^	36.0 ± 2.2vs.	33.1 ± 1.1*	2.9 ± 1.1	95.2 ± 1.0vs.	96.3 ± 0.3	0.3 ± 0.3	10.0 ± 1.0vs.	6.1 ± 0.0*	3.9 ± 1.0^a^
**SOF**	65.0 ± 2.4vs.	80.8 ± 3.1*	15.8 ± 0.7^b^	170.4 ± 4.2vs.	21.0 ± 4.5*	32.6 ± 0.0^b^	44.6 ± 2.3vs.	41.5 ± 2.5*	2.1 ± 0.8	93.4 ± 0.0vs.	94.7 ± 0.5	1.3 ± 0.5	115 ± 1.5vs.	5.50 ± 0.7*	6.0 ± 0.8^b^


# Level of difference between the two values compared. *Indicates significant difference between non-vitrified vs. vitrified values of a given comparison (p<0.05) In each row, comparisons with different letters are significantly different (p<0.05). All percentages expressed as mean ± SEM.

### Effect of culture system on developmental competence of vitrified vs. non-vitrified embryos

In table 3 the results of quality assessment of nonvitrified (Table 1) versus vitrified (Table 2) blastocysts which have been cultured under the same conditions were paired and compared.

The levels of difference (LD) between the values of each comparison were also compared (based on statistical analysis of the replicate data). As shown, irrespective of the culture conditions employed, overall quality of vitrified-warmed IVP embryos (as measured by the rates of hatching, TCN and DFI) were significantly lower than the corresponding rates of non-vitrified embryos. Moreover, by looking into the LD between vitrified/non-vitrified embryos cultured in the same condition (Table 3), the culture of embryos in SOF versus the co-culture system resulted in different LD values which approached significance for hatching, TCN and DFI percentages (15.8%, 32.6% and 6.0% vs. 9.0%, 77.6% and 3.9%, respectively).

## Discussion

Fifty years after the first report of mammalian IVP embryo (24), there are two fundamentally constructed, strongly-believed concepts about IVP media formulations; the conventional co-culturing system (8) and the more recently developed cell-free sequential media (5). Although sequential media are of more interest during the last decade (5), cryo-resistance of embryos developed in such systems seems to be unsatisfactory, particularly when compared with the related results of those embryos developed over monolayers (4, 6, 8). Given the fundamental importance of having chosen a completely efficient IVP system for both embryo development and cryo-survival, this study was carried out to investigate the possibility that restoring co-culture systems or sequential culture media can be of the same or superior credibility for in vitro embryo development and cryopreservation purposes.

The results of this study indicated somewhat new concepts regarding the interplay between an in vitro culture system, embryo development and cryoresistance of bovine embryos that differ in some points with some other studies. Taken together, obtained results indicated that despite early initial lag in development, in vitro culture of bovine presumptive zygotes in SOF versus the co-culture system significantly (p<0.05) promoted overall developmental competence of bovine IVP embryos as measured by the kinetics and percentages of blastocyst production on days 6, 7 and 8 of culture (Table 1). Moreover, cryosurvival analysis indicated that in vitro developed bovine zygotes in SOF sequential media totally devoid of helper cells provided blastocysts capable of surviving the vitrification/warming procedure in the same ratios as the co-culture system. However, final survival rates of SOF-developed embryos, as measured by total percentages of survival and hatching of vitrified/warmed embryos were significantly lower than the related rates of the blastocysts developed under the co-culture condition (p<0.05; Table 2). Quality analysis of the survived-hatched blastocysts indicated that the procedure of vitrification/ warming resulted in more deleterious effects, as measured by the TCN and the ratio of apoptotic blastomeres (DFI rate), in those blastocysts developed under sequential versus co-culture conditions. As depicted in table 1, there is no significant difference in the cleavage potential of inseminated oocytes when cultured in two completely different embryo media. Accordingly, Rizos et al. (6) showed that culture of in vitro matured/fertilized zygotes in the ewe oviduct significantly improved quality of the resulting blastocysts in terms of blastocyst development and survival post-vitrification over those produced in vitro. This would suggest that the processes of maturation and fertilization as they occur in vitro are not the main factors affecting blastocyst yield and quality (11). Indeed, it seems that the potential of the inseminated oocytes for the first mitotic division is more a reflection of previous achievement rather than the effect of culture medium (6, 11). A thorough literature search has indicated that the cleavage rate of the embryos developed under different culture conditions are commonly of a non-significant difference, and hence, one may interpret that it is actually the intrinsic quality of the oocyte itself that is the key factor in determining the first zygotic division (3, 6, 7, 9, 13).

The viability of vitrified-warmed embryos can be assessed by a number of indices; the most common is in vitro survival after a period of culture (6). Several culture systems have been used for culturing vitrified-warmed embryos in particular those have been supported by somatic cells such as oviductal epithelial cells, granulosa cells, buffalo rat liver cells or Vero cells as co-culture supports (6, 8). In a similar study and using a monolayer of granulosa cells during in vitro embryo development and/or postthawing embryo culture, it has been demonstrated that while culture in SOF lead to significantly more blastocysts compared to culture in TCM199 in the presence of a granulosa cell monolayer (GCM), blastocyst quality is inferior in terms of survival after vitrification/warming (6). Finally, they have advised that SOF-GCM (i.e., with GCM throughout the embryo development and post-warming period) is the best culture system to maximize post-warming survival. In order to acquire improved cryotolerance, the resesarchers necessitated at least a certain period of embryo culture in the presence of monolayers (6).

Although the cumulative results of current and old studies agree with the positive effects of monolayer cells for embryo cryopreservation (6, 8), the overall results of this study suggest no such need for continuing co-culture systems. The bases of this conclusion are: 1. significantly greater chance to produce more in vitro blastocysts (based on the number of in vitro matured and fertilized oocytes) using a sequential versus co-culture system as observed in this study (Table 1); 2. reasonable quality of fresh SOF-developed embryos compared to co-cultured embryos (Table 1); and 3. marginal higher quality of co-cultured versus SOF embryos which can be compensated by the higher number of blastocysts produced in SOF versus co-culture systems.

Comparison between the results of viability and apoptosis of vitrified/warmed embryos (Table 2) revealed that while the number of viable/dead supported no significant difference between these groups, the number of apoptotic cells significantly differed in the same comparison (p<0.05). This suggests that the sole assessment of viability is not a very sensitive parameter for assessment of embryo development and cryosurvivability (9). However, comparison between different quality parameters of vitrified and non-vitrified blastocysts developed under the same culture conditions (Table 3) suggests that the vitrification procedure used in this study did not have such a dramatic influence on embryo viability. However, there a significant drop in the rate of hatching and a significant increase in apoptosis rate in SOF versus co-culture derived embryos (p<0.05), implies yet a need for more studies to optimize a suitable procedure of cell-free embryo culture to achieve additional desired results.

## Conclusion

Therefore, it can be concluded that despite numerous advantages which exist for cell-free over cell-based culture media, a long-term plan should be undertaken to develop a cell free sequential media containing all the reported benefits of co-culture systems. This may be achieved by analyzing the wide range of nutrients and trophic factors released by the feeder cells in the culture milieu (25-27). Also, there is a need for a through understanding about the possible detoxifying mechanisms of the feeder cells to be mimicked using cell-free sequential media (8).
